# Non-selective Aortic Root Angiographic Contrast Injection in a Case of Anomalous Right Coronary Artery Ostium

**DOI:** 10.7759/cureus.42945

**Published:** 2023-08-04

**Authors:** Emmett Brennan, Alexa B Kahn, Abdullah Khan, Hal L Chadow, Ricardo Castillo, Rumman A Syed, Yonael Ayinalem

**Affiliations:** 1 Internal Medicine, One Brooklyn Health - Brookdale University Hospital Medical Center, Brooklyn, USA; 2 Cardiology, One Brooklyn Health - Brookdale University Hospital Medical Center, Brooklyn, USA

**Keywords:** non-selective coronary angiography, cto, non-st segment elevation myocardial infarction (nstemi), angiography, anomalous origin of right coronary artery

## Abstract

Anomalous origin of the right coronary artery (RCA) is a rare congenital cardiovascular anomaly that can pose significant diagnostic challenges during cardiac evaluation. We present a case of a 54-year-old male patient with chest pain and a syncopal episode and subsequently diagnosed with non-ST-elevated myocardial infarction (NSTEMI). Coronary angiography revealed an anomalous origin of the RCA, making it difficult to precisely locate the artery's point of origin with selective contrast injection. During coronary angiography, the use of aortic root non-selective angiographic contrast injection aided in localizing the RCA ostium.

Our case highlights the clinical significance of aortic contrast injection as a valuable and safe adjunctive technique in cases of anomalous coronary artery origins. Early detection and precise localization of such anomalies are essential for effective treatment planning and improved patient outcomes. Further studies may help validate the utility of aortic contrast injection in similar cases, thereby enhancing diagnostic accuracy and patient care in the management of anomalous coronary artery ostium.

## Introduction

Anomalous coronary artery origin is a rare but clinically significant congenital anomaly that involves the abnormal origin or course of one or more coronary arteries from the aorta or other non-standard locations. These anomalies affect approximately one percent of the general population [1] While many individuals with such anomalies may remain asymptomatic and are detected incidentally on imaging or coronary angiography, there is also a well-documented association between anomalous coronary artery origin and severe cardiovascular events, including sudden cardiac death, myocardial infarction, and arrhythmias [2,3].

In this case report, we present a compelling clinical encounter of a patient diagnosed with a non-ST-segment elevated myocardial infarction (NSTEMI) and who was subsequently found to have an anomalous origin of the right coronary artery (RCA) on cardiac catheterization. The significance of this anomaly lies not only in its rarity but also in the potentially life-threatening consequences it poses and the technical challenges it can pose during angiography.

By elucidating the clinical presentation, diagnostic workup, and subsequent management of this patient, we seek to contribute valuable insights to the medical community. Our objective is to enhance awareness of anomalous coronary artery origins among healthcare professionals and the potential role of non-selective angiographic contrast injection in detecting such an anomaly.

## Case presentation

A 48-year-old gentleman with a past medical history of hypertension and non-compliance with his medication regimen presented via ambulance with a 12-hour history of chest pain. The pain was described as substernal, radiating to the left shoulder, and 10/10 in intensity. The patient also had associated diaphoresis, and a five-minute loss of consciousness was reported. He was given sublingual nitroglycerin and 324mg aspirin in the field with improvement in his pain. On arrival at the hospital, his blood pressure was 159/105, and his heart rate was 89. He was given a loading dose of ticagrelor and started on nitroglycerin infusion for suspected Acute Coronary syndrome. He was admitted to the Coronary Care unit.

**Figure 1:**
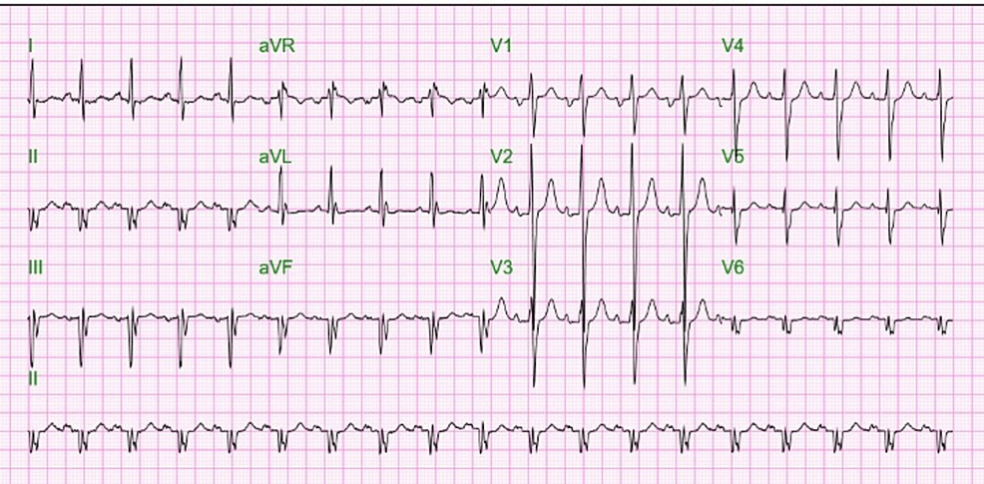
Electrocardiogram showing Q waves in the inferior leads and V2 and tall R in V2

**Figure 2:**
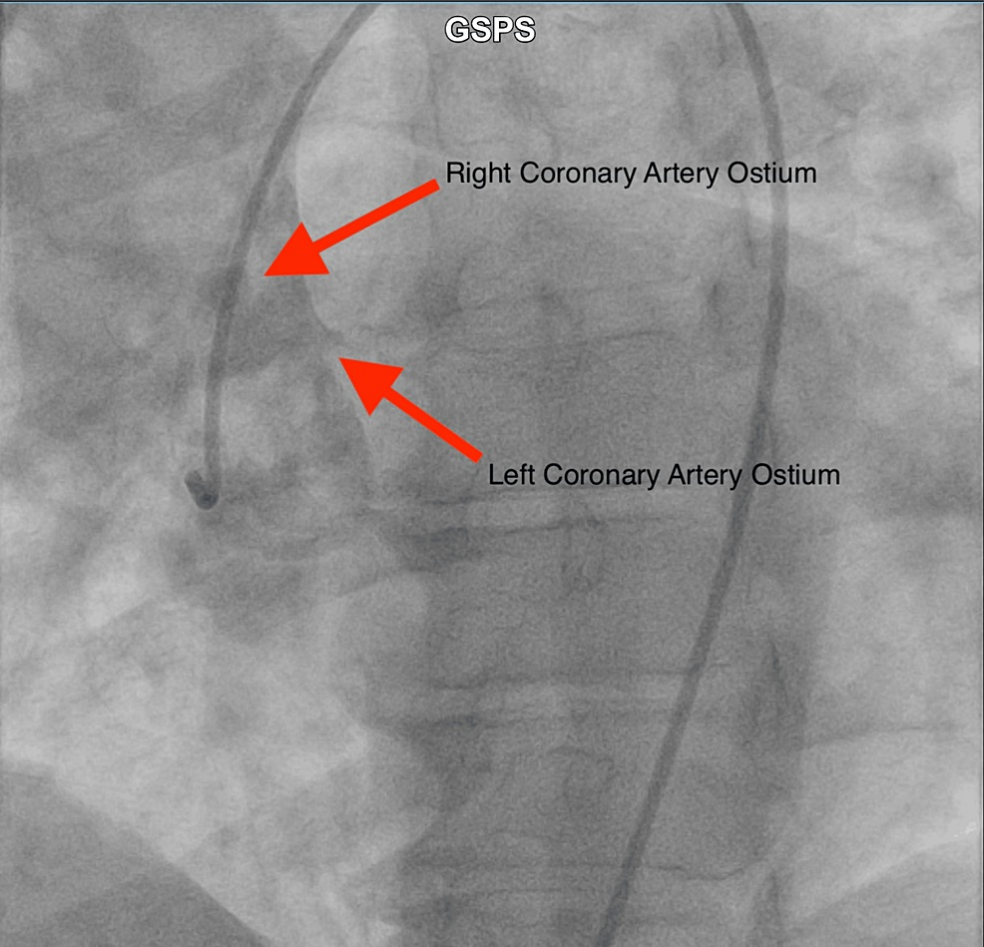
Non-selective aortic root contrast injection revealing high take of the right coronary artery ostium (arrowhead)

Initial ECG was indicative of an infero-posterolateral NSTEMI with Q waves in the inferior leads and V2 and tall R in V2 (Figure 1). Initial troponin was 6.67 ng/mL, and NT pro-BNP was 5,260 pg/mL. Peak troponin was identified at 50.3 ng/mL. The patient was brought for cardiac catheterization for non-ST segment elevation myocardial infarction. Initial cardiac catheterization revealed normal left main coronary artery, 75% stenosis in the mid-third of the first diagonal branch, and 100% stenosis of the mid circumflex after the origin of the first obtuse marginal branch with TIMI grade 0 flow. The RCA could not be selectively cannulated after trialing multiple catheters. Repeat heart catheterization via the right femoral approach was performed. Non-selective contrast injection revealed the origin of the RCA high off the aorta (Figure 2).

**Figure 3:**
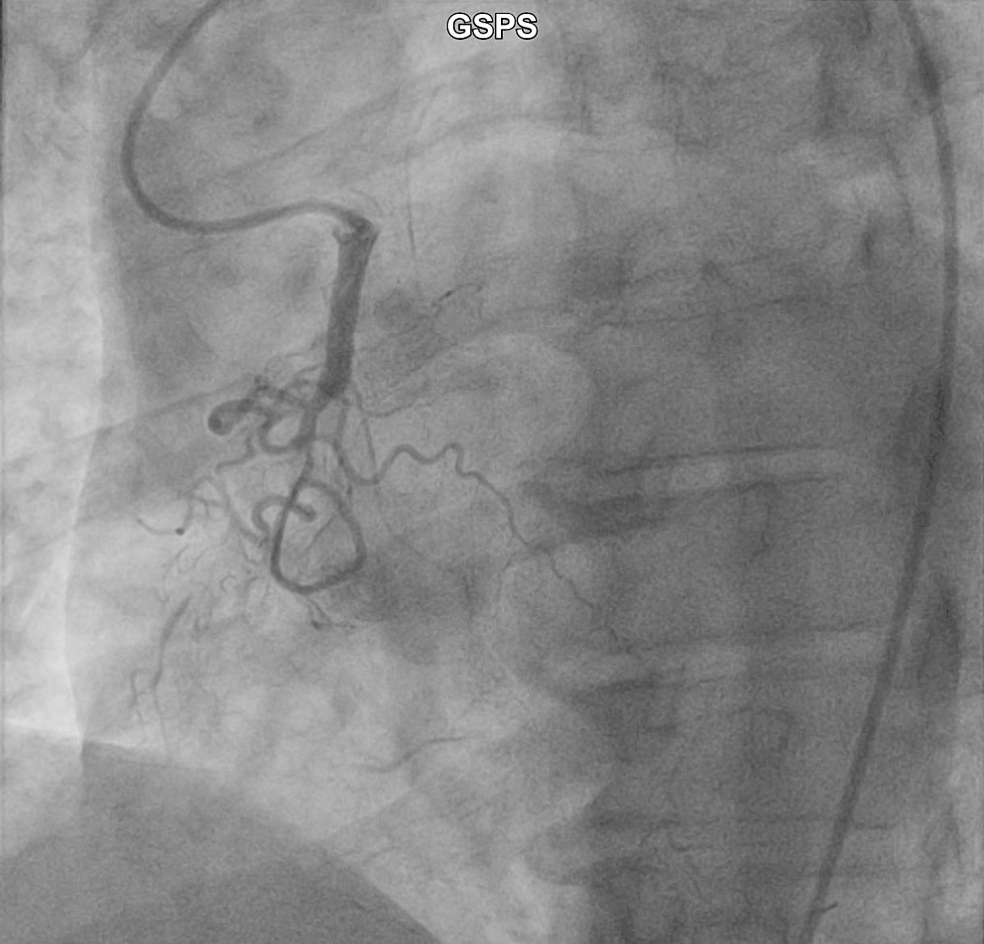
Selective contrast injection of the engaged right coronary artery revealing a chronic total occlusion of the vessel

The RCA was engaged using a 6 French Amplatz Right 1 diagnostic catheter (Figure 3) and there was 100% occlusion in the mid-segment. 100% occlusion with peri-obstructive collaterals noted. The intervention was unsuccessful. A transthoracic echocardiogram reported left ventricular dilation and mildly reduced systolic function with an ejection fraction of 45% to 50%. There was akinesis of the basal inferoseptal, basal inferior, and basal-mid inferolateral walls, and Doppler parameters were consistent with abnormal left ventricular relaxation (grade 1 diastolic dysfunction). 

The patient was diagnosed with triple vessel disease. The electrophysiology service was consulted due to his loss of consciousness on presentation, and this was attributed to a vasovagal episode secondary to the NSTEMI. The patient was discharged on dual antiplatelet therapy, high-intensity statin therapy and Goal-directed medical therapy for Heart failure with mid-range Ejection fraction 9 HFmrEF. At the clinic follow-up one-month post-discharge, he was chest pain-free and was maintaining good compliance with his goal-directed medical therapy.

## Discussion

Anomalous origin of the RCA is a rare congenital abnormality characterized by an aberrant ostium location, deviating from the typical anatomical course. One study reported a prevalence for an anomalous RCA origin of approximately 1% in a large series of coronary anomalies involving over 126,000 patients undergoing coronary arteriography [1]]. The clinical significance of an anomalous coronary artery can vary depending on the location of the origin and course of the vessel. The anomalous RCA variant taking an intramural course between the aorta and pulmonary artery, or right ventricular outflow tract is of particular concern. This anatomical arrangement can lead to extrinsic compression of the coronary artery during periods of increased cardiac output or exertion, impairing coronary blood flow and causing myocardial ischemia and the potential for sudden cardiac events [4,5].

The high take-off origin of the RCA has been defined as an RCA origin that is 5-10mm above the sinotubular junction. It has been described in approximately 0.019%-0.17% of autopsy studies [6]. The case we present describes a high take-off RCA with an ostium visualized above the coronary cusps in a gentleman who presented with an NSTEMI. Patients with a high take-off RCA may remain asymptomatic, and the finding is detected incidentally in imaging studies or during surgical procedures [7,8]. Occasionally cases of high take-off of the RCA have been linked with cases of angina, myocardial infarction, and sudden cardiac death [9]. A high take-off RCA typically follows a longer course of the vessel and may have a slit-like ostium. Both factors have been previously posited to have a potential role in the development of ischemia [5,8].

The utility of aortic root non-selective contrast injection in cases of anomalous coronary arteries has been recognized in the literature. Aortic root non-selective contrast injection can be particularly useful in localizing anomalous coronary artery origins and differentiating them from other structures in the vicinity of the Ostia. A single-center study utilized this approach when the selective engagement of the RCA proved difficult during a study on coronary interventions in cases of anomalous RCA origin [10]. In the case we described, selective engagement of the vessel proved challenging. A non-selective aortic contrast root injection was employed in order to visualize the ostium of the RCA and aid in engaging the vessel. In cases of anomalous RCA ostium, traditional selective angiography may fail to provide sufficient clarity due to the unusual origin of the artery. In such instances, aortic root contrast injection emerges as a valuable adjunctive technique to enhance visualization and delineate the course of the anomalous RCA.

This clinical case demonstrates the finding of an anomalous high take-off RCA origin in a gentleman who presented with NSTEMI. The case also demonstrates the potential utility of aortic root non-selective contrast injection in identifying the ostium of the vessel in such cases.

## Conclusions

Anomalous coronary artery origin, a rare congenital anomaly involving aberrant positioning or course of one or more coronary arteries, has been recognized as an important clinical entity with potential life-threatening implications. In this case report, we present a case of a patient who presented as an NSTEMI and was found to have a high take-off RCA ostium during coronary angiography. This case report highlights the potential role of the use of a non-selective Aortic root contrast injection for identifying a rare anomalous high take-off RCA ostium in cases where the coronary artery ostium proves challenging to locate and engage.

## References

[1] Angelini P, Velasco JA, Flamm S (2002). Coronary anomalies: incidence, pathophysiology, and clinical relevance. Circulation.

[2] Yamanaka O, Hobbs RE (1990). Coronary artery anomalies in 126,595 patients undergoing coronary arteriography. Cathet Cardiovasc Diagn.

[3] Cheitlin MD, De Castro CM, McAllister HA (1974). Sudden death as a complication of anomalous left coronary origin from the anterior sinus of Valsalva, a not-so-minor congenital anomaly. Circulation.

[4] Molossi S, Martínez-Bravo LE, Mery CM (2019). Anomalous aortic origin of a coronary artery. Methodist Debakey Cardiovasc J.

[5] Greet B, Quinones A, Srichai M, Bangalore S, Roswell RO (2012). Anomalous right coronary artery and sudden cardiac death. Circ Arrhythm Electrophysiol.

[6] Deng X, Huang P, Chen W, Yang X, Liu Q, Xiao Y, He C (2017). An incidental encounter of a rare high take-off right coronary artery: a case report. Medicine (Baltimore).

[7] Wang SP, Jao YT, Han SC (2008). Acute coronary syndrome due to high aortocoronary junction of the right coronary artery: the value of multislice CT. Int J Cardiol.

[8] Alpaslan M, Onrat E (2002). Anomalous origin of right coronary artery above the sinus of Valsalva: observation by transthoracic echocardiography. J Am Soc Echocardiogr.

[9] Tarhan A, Kehlibar T, Yilmaz M, Arslan Y, Pancaroglu C, Yigit S, Ozler A (2007). Right coronary artery with high takeoff. Ann Thorac Surg.

[10] Uthayakumaran K, Subban V, Lakshmanan A (2014). Coronary intervention in anomalous origin of the right coronary artery (ARCA) from the left sinus of valsalva (LSOV): a single center experience. Indian Heart J.

